# Supplementary material for: UBXN3B positively regulates STING-mediated antiviral immune responses
Source: Nat Commun. 2018 Jun 13;9:2329. doi: 10.1038/s41467-018-04759-8 (PMC5998066; doi:10.1038/s41467-018-04759-8)

UBXN3B positively regulates STING-mediated antiviral immune responses

Yang et al.

Supplementary Figures 1-11

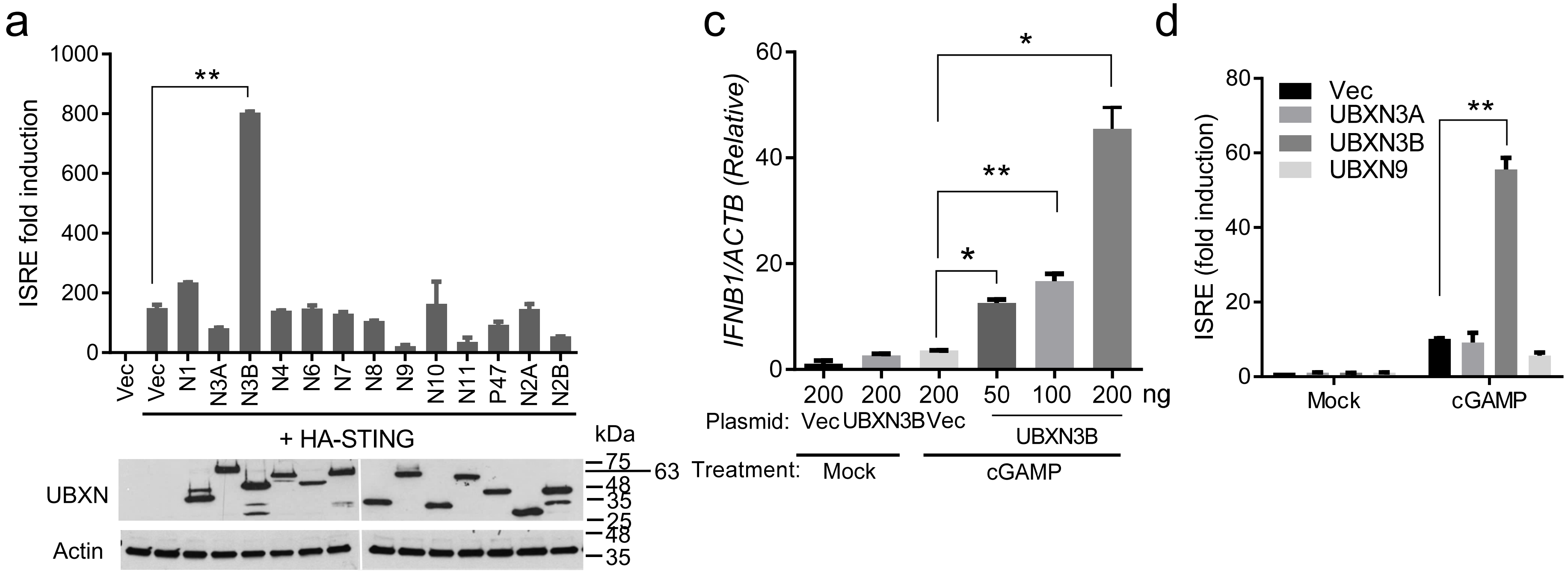

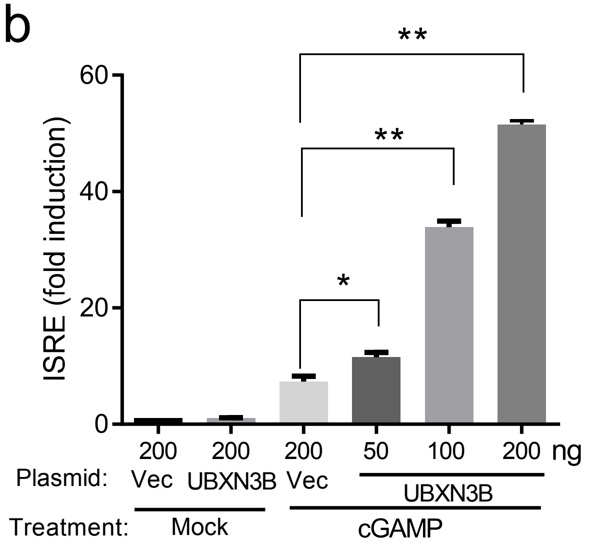

Supplementary Figure 1 UBXN3B potentiates STING-mediated type I IFN responses.

(a) Quantification of ISRE luciferase activity in HEK293 cells transfected with 100ng vector (Vec) and individual FLAG-UBXN plasmids without/with 100ng HA-STING. The immunoblots under the bar graph show the FLAG-UBXNs protein expression levels. Actin is a housekeeping protein control. **(b)** and **(d)** Quantification of ISRE luciferase activity in HEK293-STING cells transfected with vector or (b) increasing doses of FLAG-UBXN3B, (d) 100ng of indicated FLAG-UBXNs and then transfected with 4μg/ml cGAMP. **(c)** qPCR quantification of *IFNB1* mRNA expression in (b). The results are presented as mean ± s.e.m of fold induction over vector of Mock. *, $P<0.05$; **, $P<0.01$ (unpaired Student's t-test), N=2. Three biological replicates were pooled for qPCR (N=2 per treatment). The results shown are representative of 2 independent experiments.

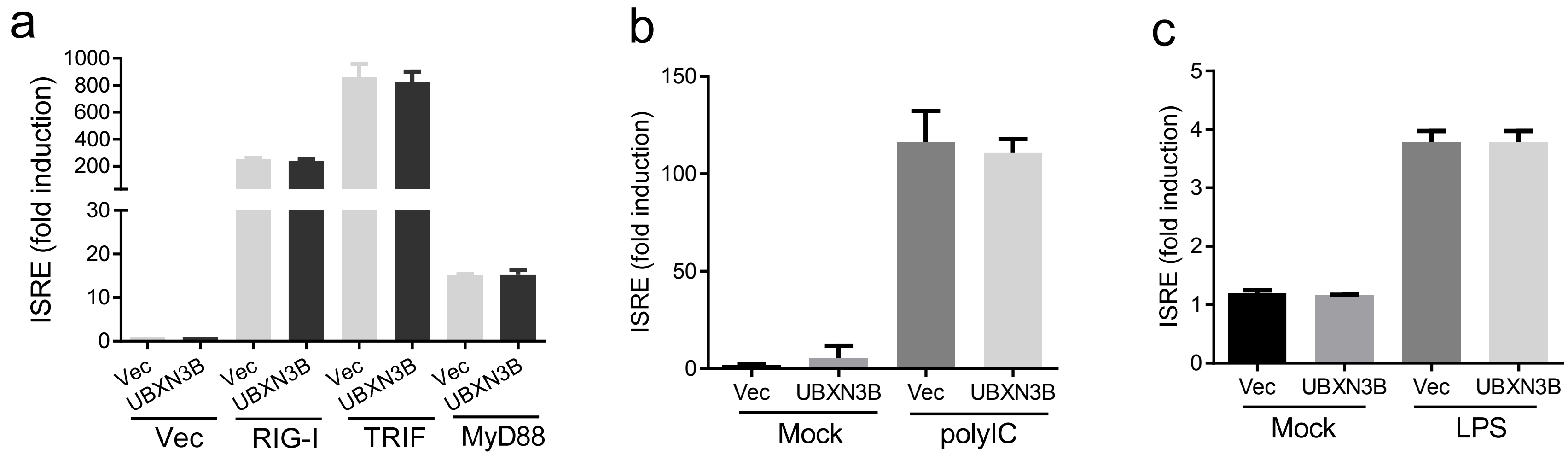

Supplementary Figure 2 UBXN3B does not potentiate type I IFN induction by RLRs or TLRs.

(a-c) Quantification of ISRE luciferase activity by dual luciferase assay **(a)** in HEK293T cells transfected with 50ng of empty vector (Vec) or FLAG-UBXN3B together with ΔRIG-I (constitutively active form), TRIF or MyD88, **(b)** in HEK293T cells transfected with 100ng of FLAG-TLR3 (specifically localizes to the plasma membrane) and vector or FLAG-UBXN3B, followed by treatment with 10μg/ml of polyIC, and **(c)** in TLR4/MD2/CD14-expressing HEK293 cells transfected with vector or FLAG-UBXN3B and then treated with 50ng/ml of lipopolysaccharide (LPS) for 8h. The results are expressed as mean + s.e.m of fold induction over vector from 2 biological replicates, 2 technical replicates/each. P>0.05 (unpaired Student's t-test). The results shown are representative of 2 independent experiments.

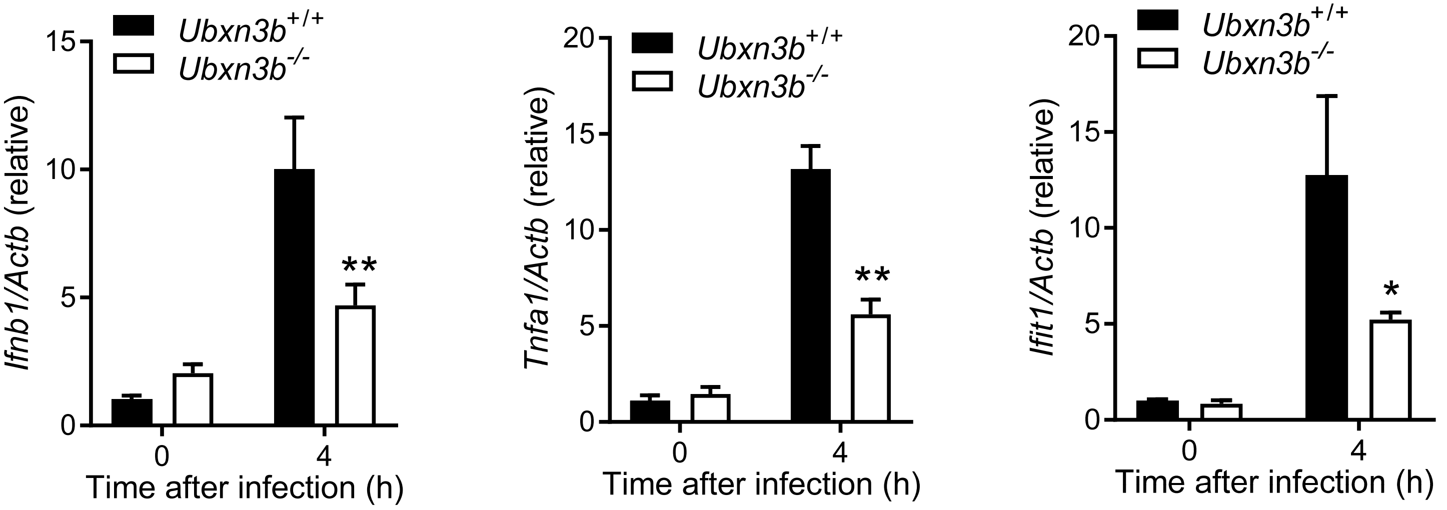

Supplementary Figure 3 UBXN3B is critical for HSV-1 induced immune responses *in vivo*.

qPCR quantification of selected immune gene mRNA expression in leukocytes isolated from HSV-1 infected mice. Bars: mean + s.e.m. N=5 mice per genotype, per censored time-point; *, $p<0.05$; **, $p<0.01$ (non-parametric Man-Whitney t-test).

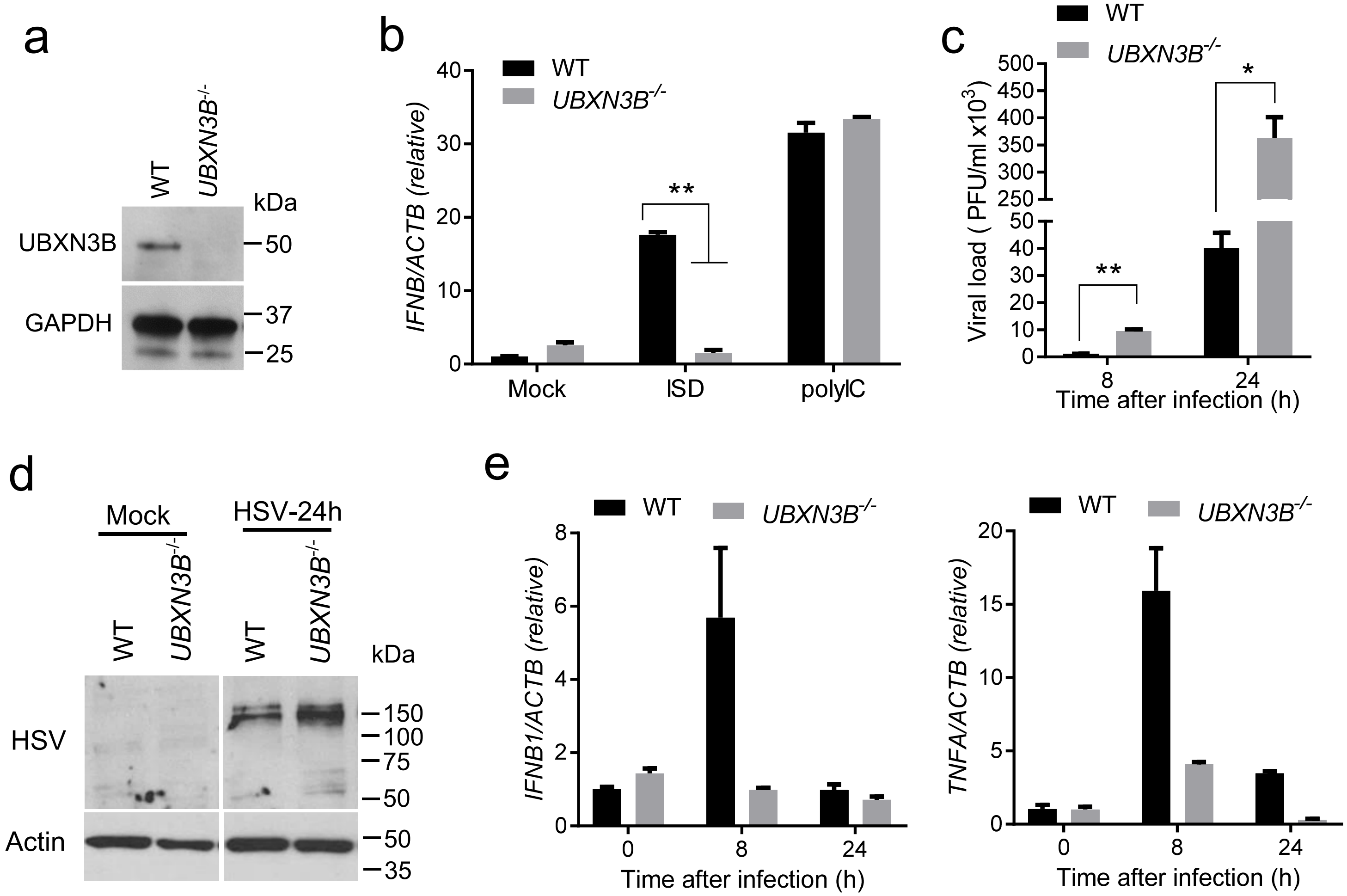

Supplementary Figure 4 UBXN3B is critical for control of HSV-1 and antiviral immune responses in human cells.

(a) The human UBXN3B expression in H1975 cells was abolished via the CRISPR-Cas9 technology. The immunoblots show knockout efficacy of two independent guide-RNA targets. **(b)** qPCR quantification of *IFNB1* mRNA induction by ISD and polyIC. WT and *UBXN3B$^{-/-}$* H1975 cells were transfected with 4μg/ml ISD or 20μg/ml polyIC for 24h. **(c-e)** Cells were infected with HSV-1 (MOI=0.5) for the indicated time. **(c)** Viral titers in the cell culture media (N=3 per genotype per time point), PFU: plaque forming units. **(d)** The immunoblots show intracellular HSV-1 protein expression. **(e)** qPCR quantification of selected immune gene mRNA expression. GAPDH and Actin are housekeeping gene controls for protein loading in (a) and (d). Bars: mean + s.e.m. *, P<0.05; **, P<0.01 (unpaired Student's t-test). Three biological replicates were pooled for qPCR (N=2 per genotype per time point). The results are representative of 2 independent experiments.

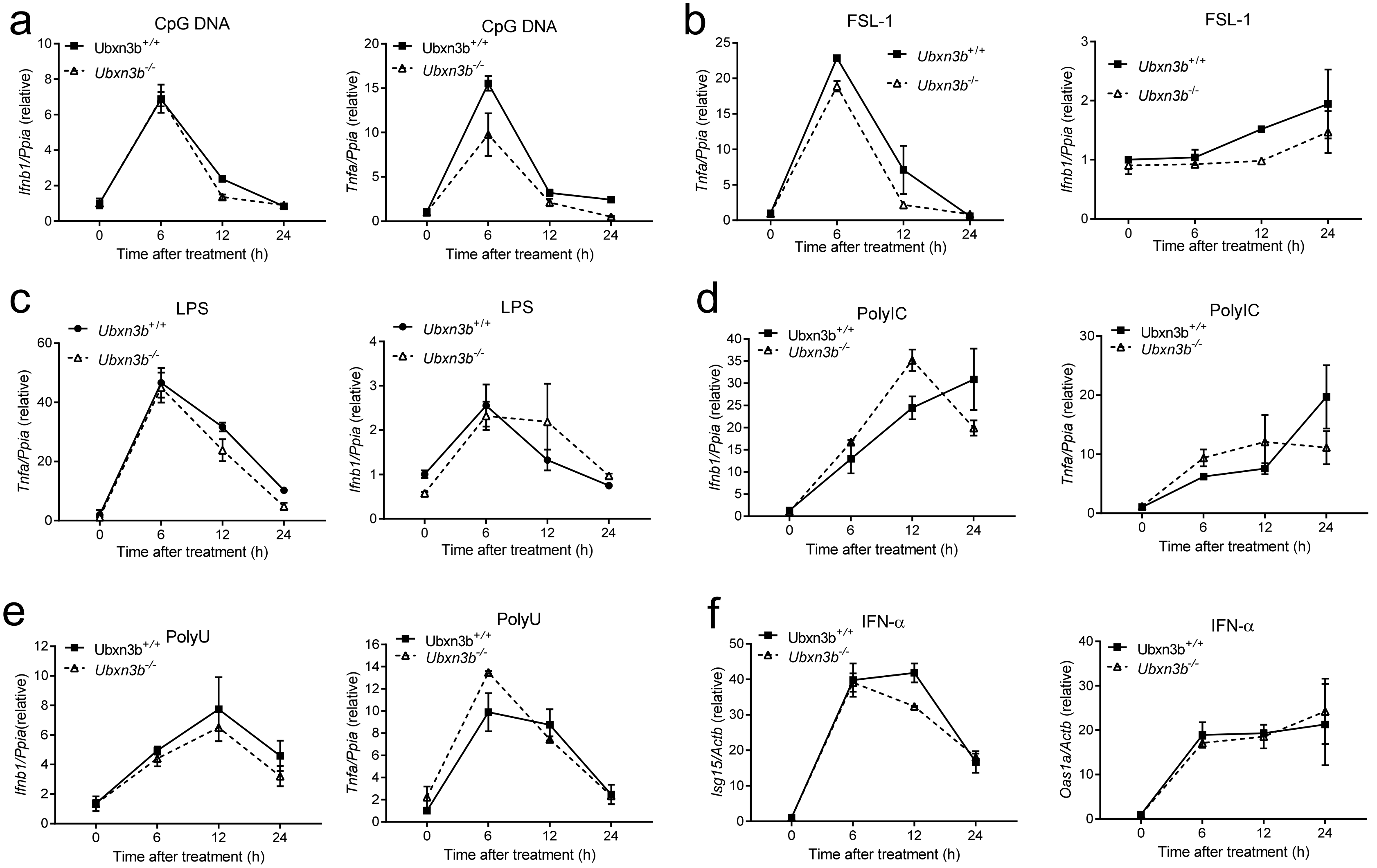

Supplementary Figure 5 UBXN3B is dispensable for TLR and JAK-STAT signaling in primary mouse DCs.

qPCR quantification of *Ifnb1* and *Tnfa* mRNA expression in Ubxn3b$^{+/+}$ and *Ubxn3b*$^{-/-}$ BM-DCs that had been **(a)** transfected with 2μM of TLR9 ligand (CpG DNA), **(b)** treated with 100ng/ml of TLR2/TLR6 ligand (FSL-1), **(c)** treated with 100ng/ml of TLR4 ligand (LPS), **(d)** transfected with 20μg/ml of MDA5 ligand (heavy molecular weight PolyIC), and **(e)** transfected with 10μg/ml of TLR7 ligand (single stranded PolyU). **(f)** qPCR quantification of interferon induced genes *Isg15* and *Oas1a* mRNA expression in BM-DCs treated with 0.3ng/ml of recombinant mouse IFN-α for the indicated time. Data points: mean ± s.e.m. Two biological replicates were pooled for qPCR (N=2 per genotype per time point). The results are representative of 2 independent experiments.

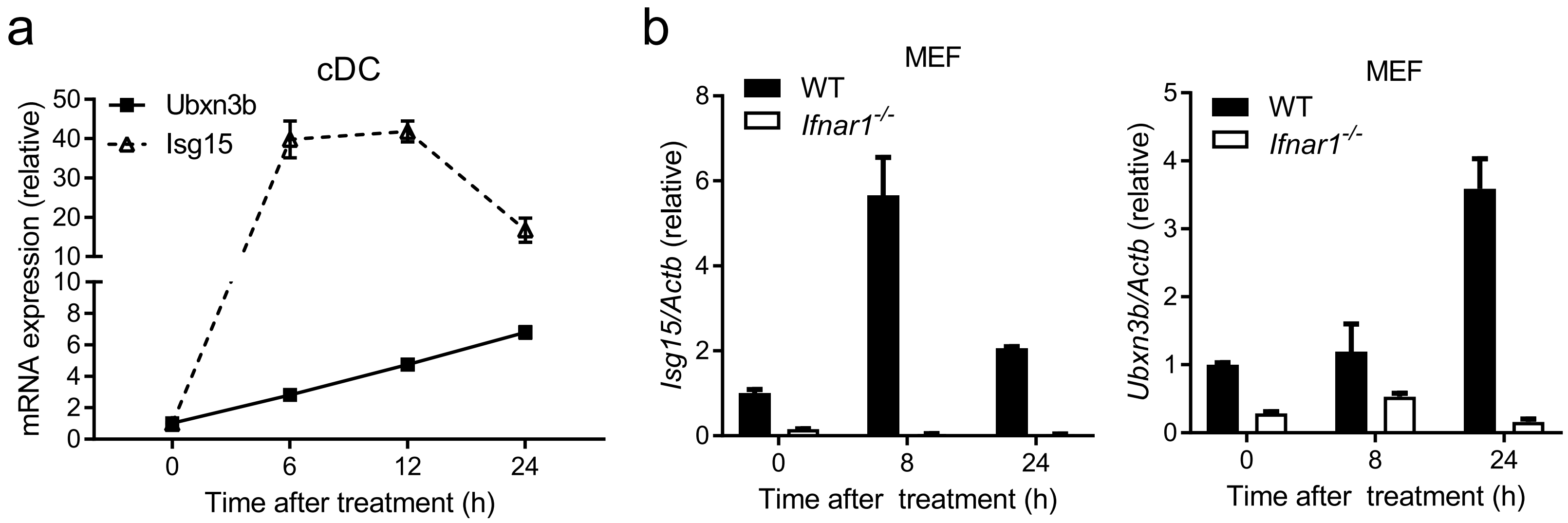

Supplementary Figure 6 UBXN3B is a type I interferon induced gene (ISG) .

qPCR quantification of interferon induced genes *Isg15* and *Ubxn3b* mRNA expression in (**a**) BM-DCs treated with 0.3 ng/ml , and (**b**) WT or *Ifnar* $^{-/-}$ MEFs treated with 0.2 ng/ml of recombinant mouse IFN-α for the indicated time. Data points and bars: mean ± s.e.m. Two biological replicates were pooled for qPCR (N=2 per genotype per time point). The results are representative of 2 independent experiments.

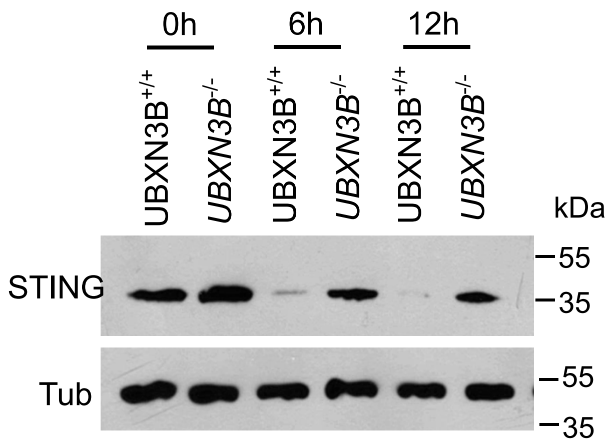

Supplementary Figure7 HSV-1-induced STING degradation is inhibited in *UBXN3B*$^{-/-}$ human cells.

H1975 cells were infected with HSV-1 at a MOI 0.5 for the indicate time. STING was detected using an anti-STING antibody. Tubulin is a house keeping control. The results are representative of 3 independent experiments.

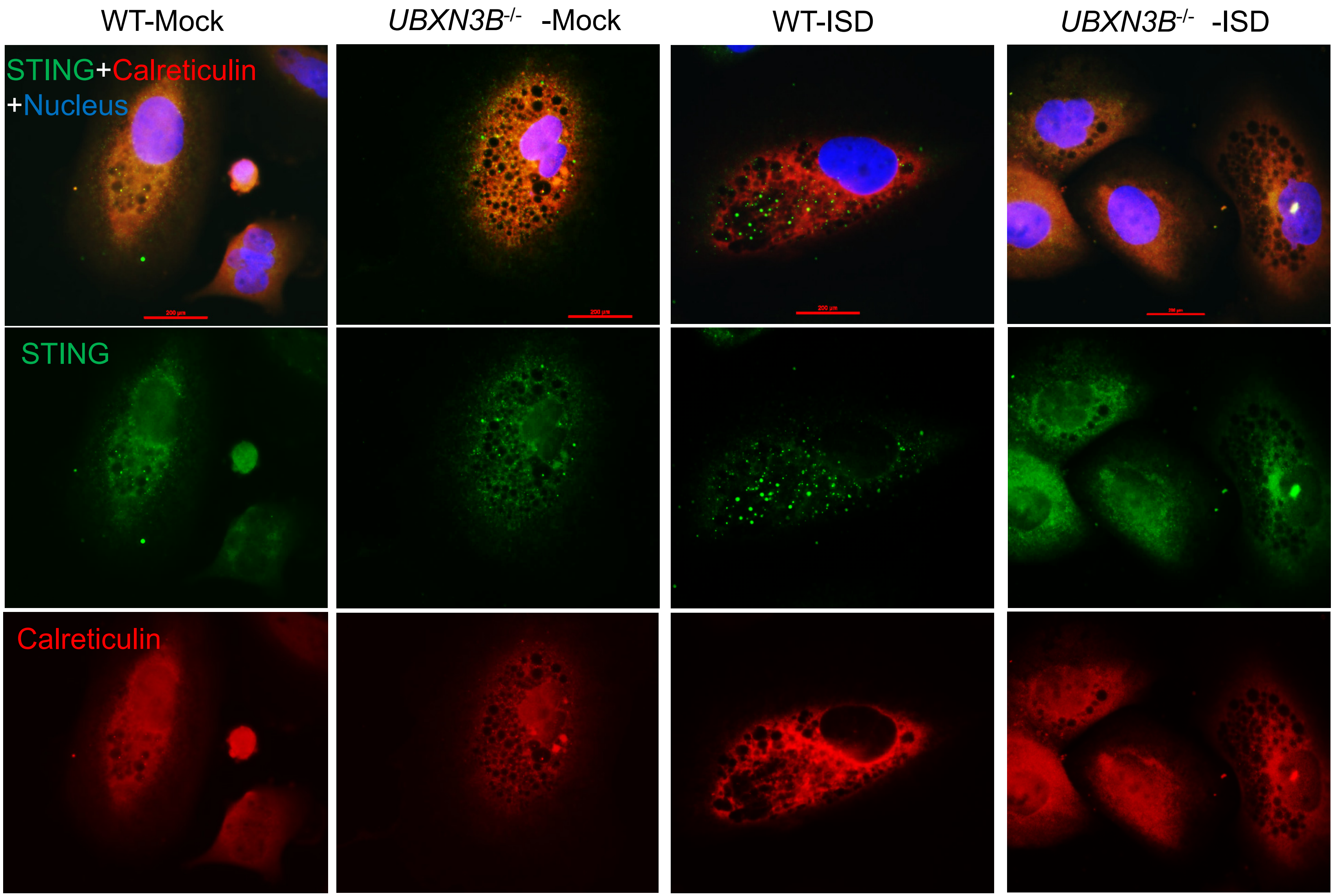

Supplementary Figure 8 STING trafficking is impaired in *UBXN3B*$^{-/-}$ cells.

Immunofluorescent staining of STING and an ER resident protein calreticulin in H1975 cells treated without (mock) or with 8μg/ml of ISD for 3 hrs. The nuclei were stained by DAPI. The images were acquired with an inverted Nikon Eclipse Ti fluorescence microscope. Scale bar, 200 μm. The results are representative of 2 independent experiments.

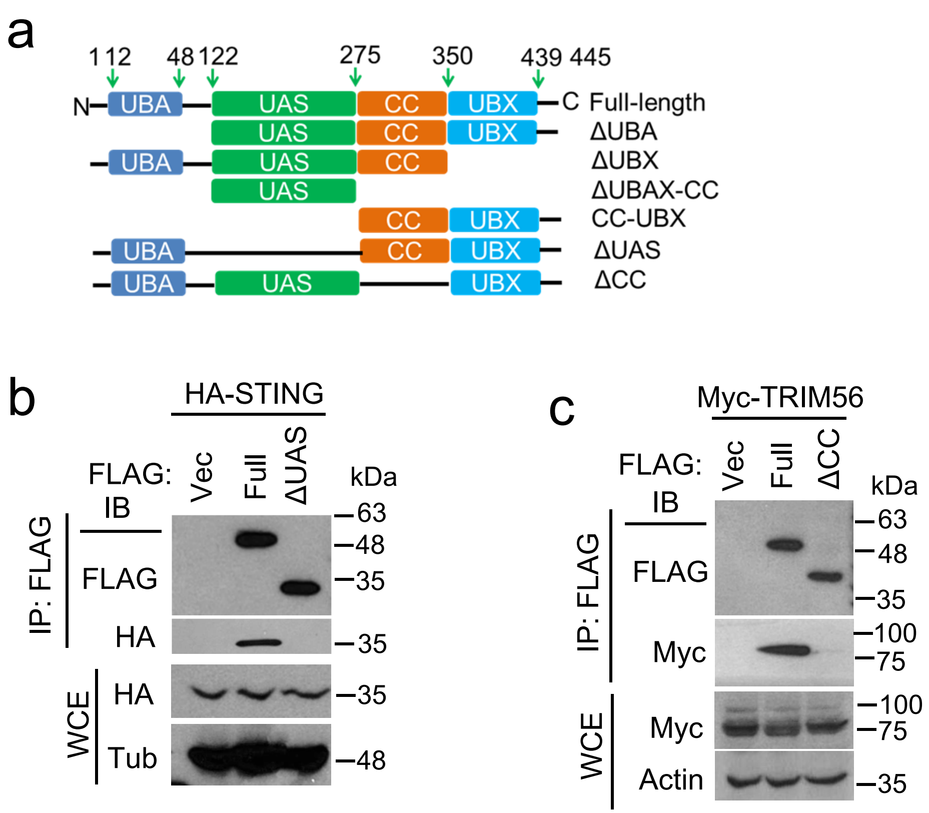

Supplementary Figure 9 UBXN3B interacts with STING via its UAS and TRIM56 via its coiled coil (CC) domain respectively.

(a) Schematic diagram of UBXN3B functional domains. Co-immunoprecipitation (co-IP) of FLAG-UBXN full-length (full) or deletion mutant (ΔUAS, ΔCC) with **(b)** HA-STING or **(c)** Myc-TRIM56 with anti-FLAG magnetic beads, followed by immunoblotting (IB) with an FLAG (UBXN), Myc (TRIM56) and HA (STING) antibody. Actin and Tubulin (Tub) are house keeping controls. The results are representative of 2 independent experiments.

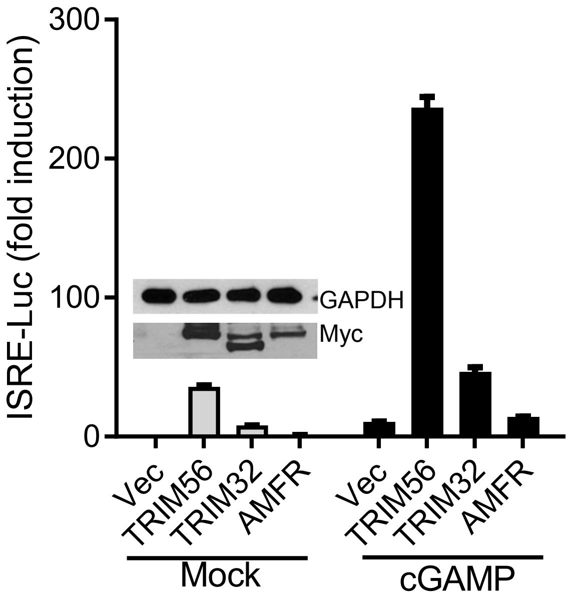

Supplementary Figure 10 Overexpression of TRIM32 and TRIM56 activates STING –dependent IFN-I response.

HEK293T-STING cells were transfected with vector, Myc-TRIM32, TRIM56 or AMFR plasmid. 16h later, the cells were transfected with 8μg/ml cGAMP together with luciferase reporter plasmid pGL3-ISRE and an internal control, pRL-TK. The luciferase activity was assessed using a Dual Glow luciferase kit (Promega) 24h after second transfection . The results are expressed as mean + s.e.m of fold induction over Mock treated vector. N=2 biological replicates. The insert shows immunoblots of Myc-tagged protein expression and a housekeeping control GAPDH. The results are representative of 2 independent experiments.

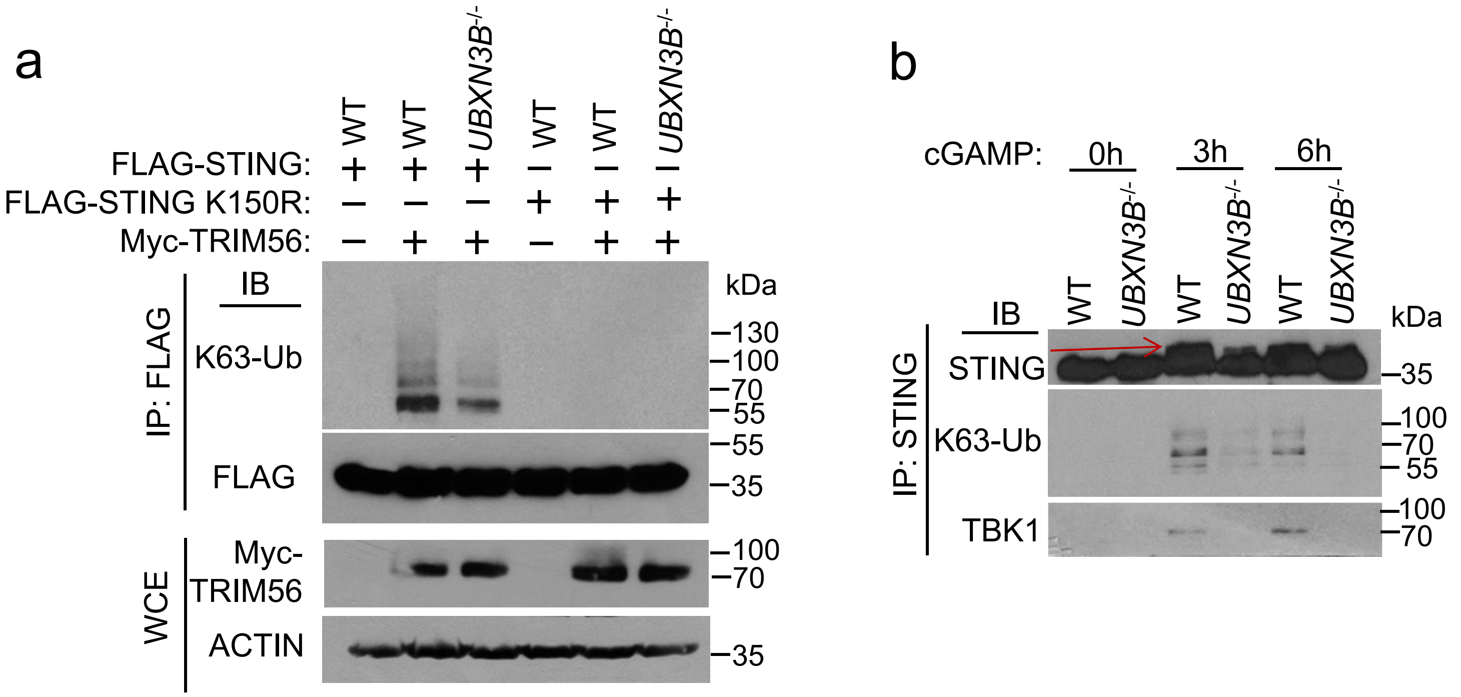

Supplementary Figure 11 UBXN3B regulates STING ubiquitination by TRIM56.

(a) WT and *UBXN3B*$^{-/-}$ HEK293T cells were transfected with the indicated combinations of plasmids. FLAG-STING or K150R mutant was immunoprecipitated (IP) with anti-FLAG magnetic beads. The proteins in IP and whole cell extract (WCE) were immunoblotted by an anti-K63-linked polyubiquitin, anti-Myc (TRIM56) and anti-FLAG (STING) antibody respectively. **(b)** STING was precipitated with a rabbit anti-STING antibody from trophoblasts transfected with 8µg/ml of cGAMP for the indicated time. The arrow points to phosphorylated STING. The proteins in IP were immunoblotted with an mouse anti- STING, anti-Ub and TBK1 antibody. Actin is a house keeping control. The results are representative of 2 independent experiments.

UBXN3B positively regulates STING-mediated antiviral immune responses

Yang et al.

The Supplementary Information contains full-length gels and molecular weights for the immunoblots in the Figures and Supplementary Figures

Fig. 1a

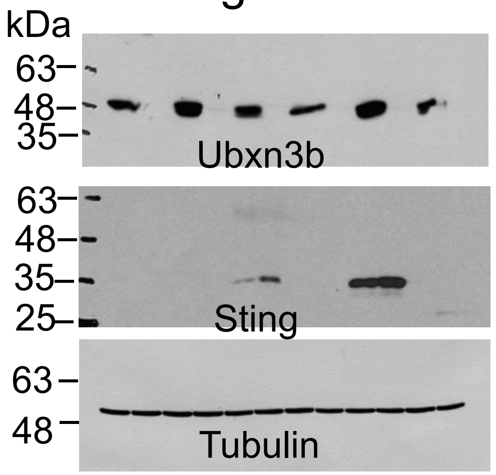

Fig. 2b

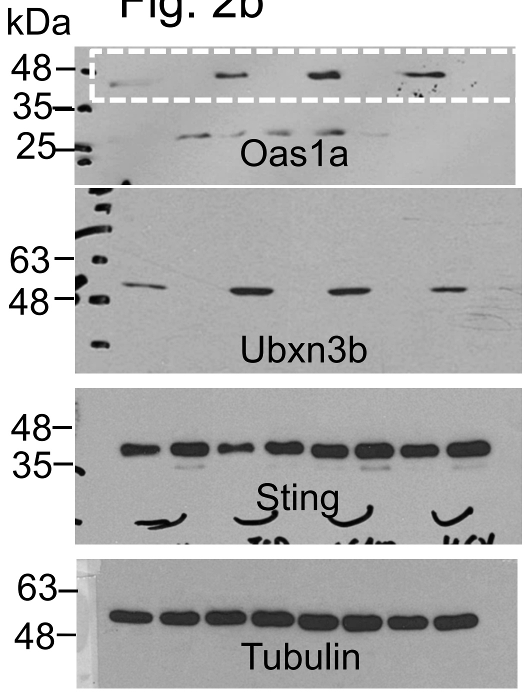

Fig. 2e

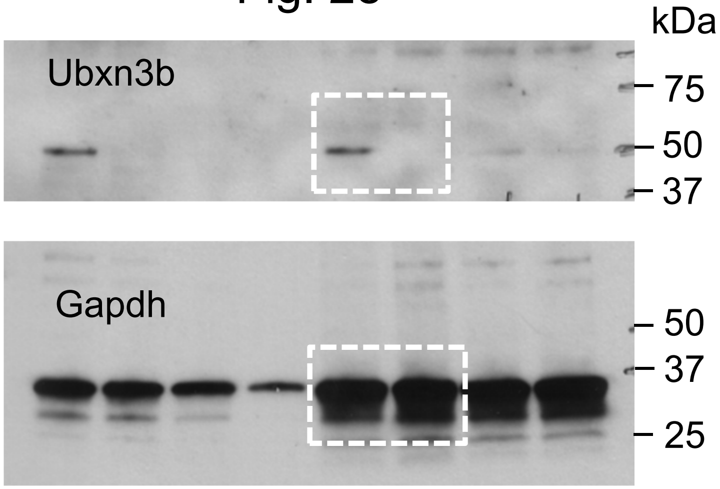

Fig. 2h

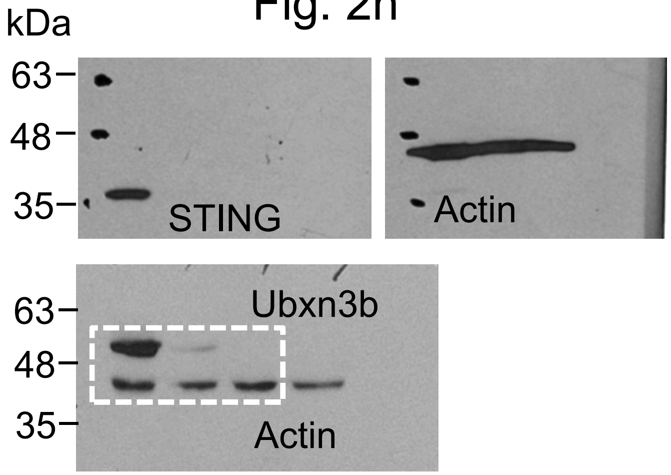

Fig. 4a

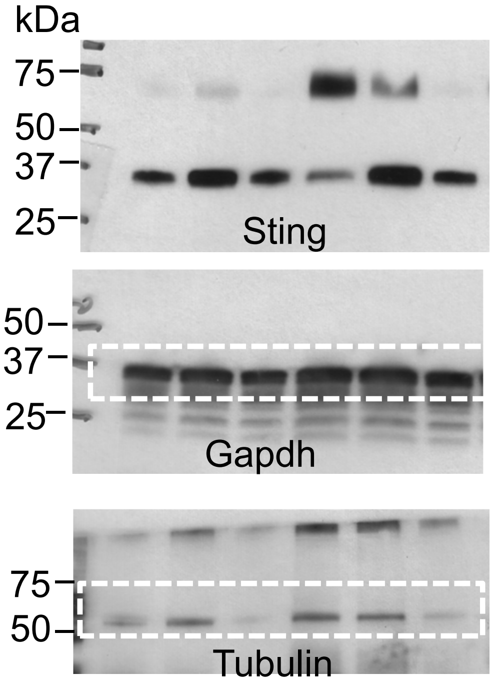
kDa
75
50
37
25
Sting
50
37
25
Gapdh
75
50
Tubulin

Fig. 4b

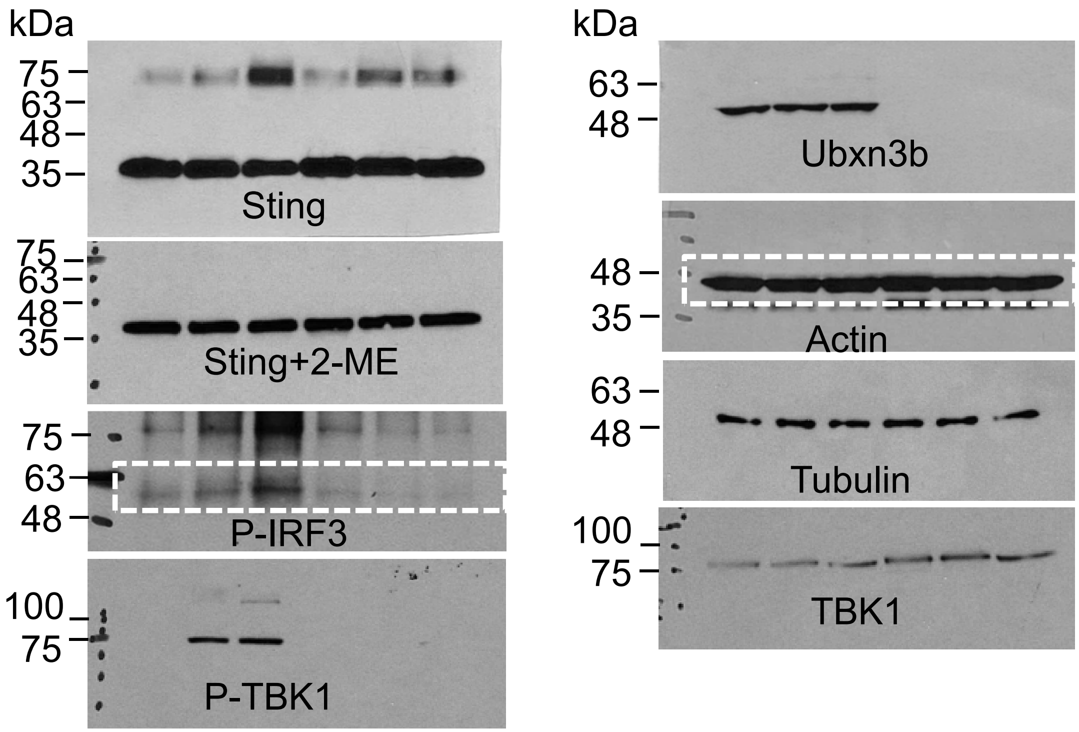
kDa
75
63
48
35
Sting
75
63
48
35
Sting+2-ME
75
63
48
P-IRF3
100
75
P-TBK1
kDa
63
48
Ubxn3b
48
35
Actin
63
48
Tubulin
100
75
TBK1

Fig. 4d

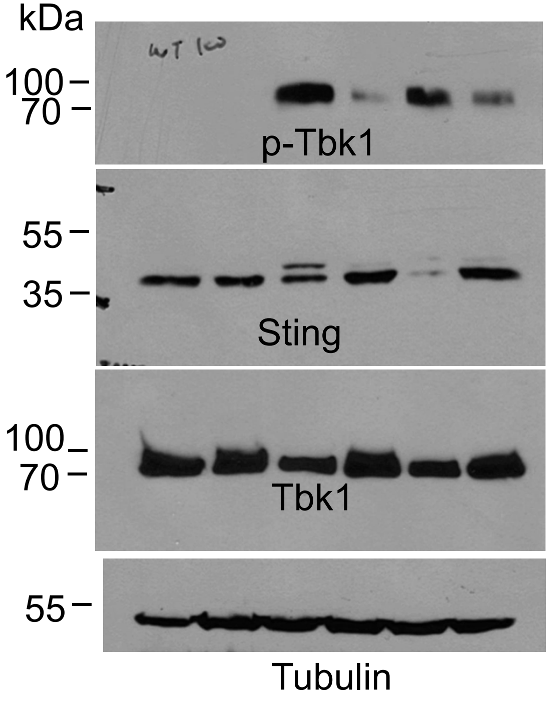

Fig. 4e

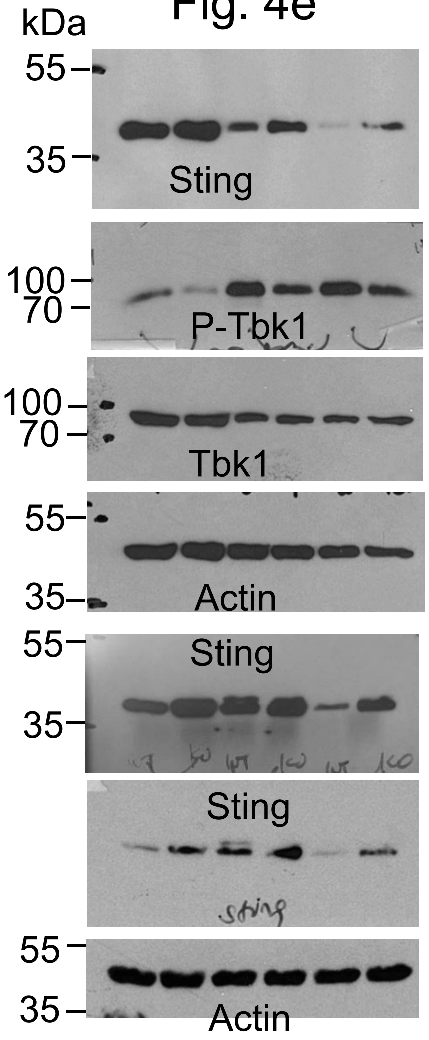

Fig. 4f

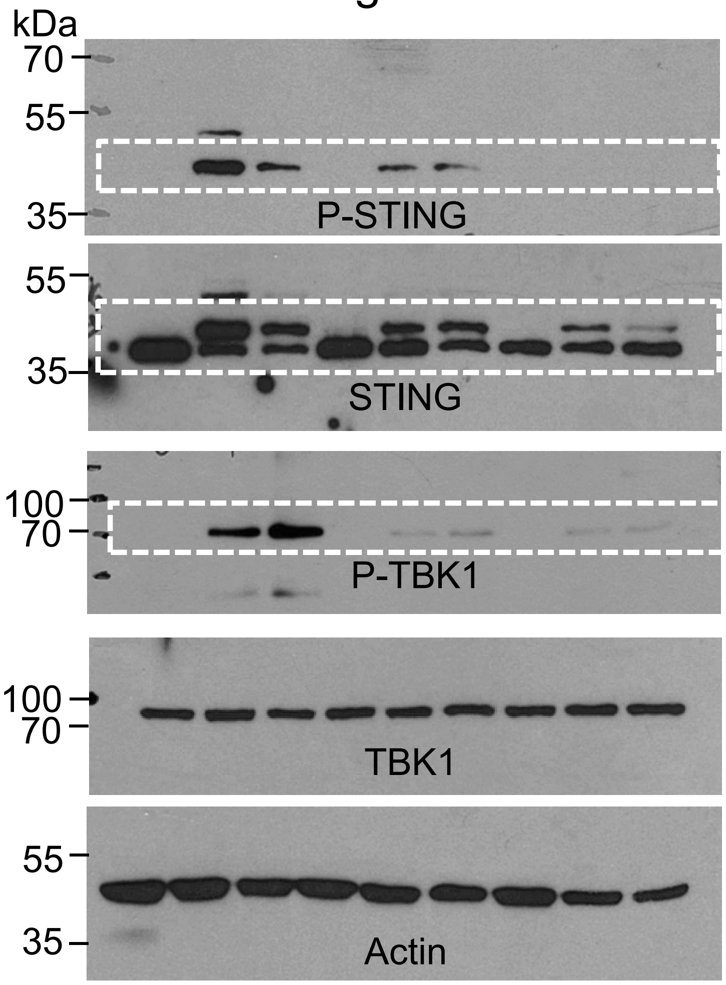

Fig. 5a

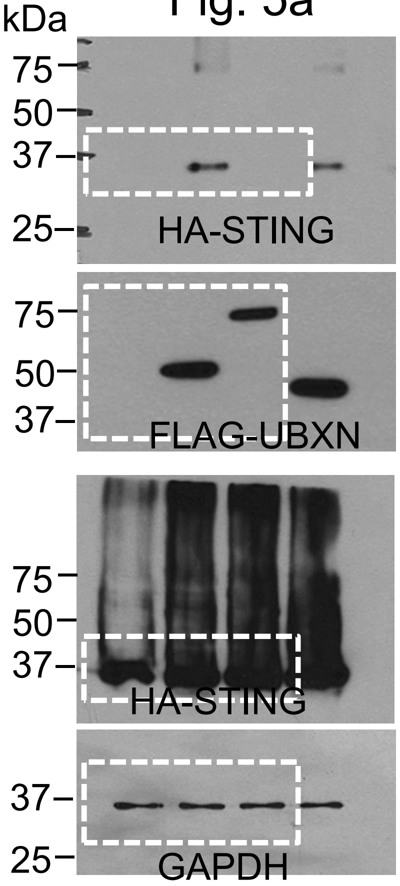

Fig. 5d

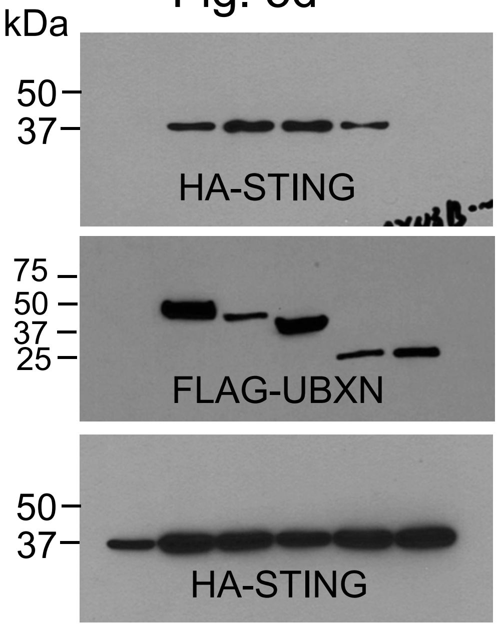

Fig. 5e

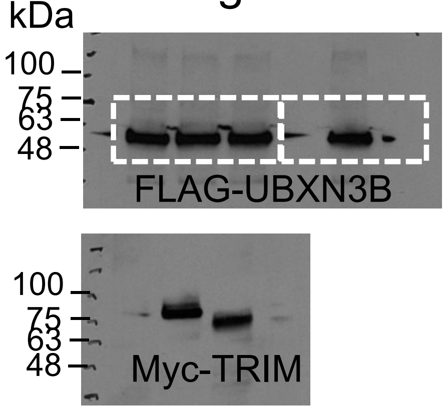

Fig. 5f

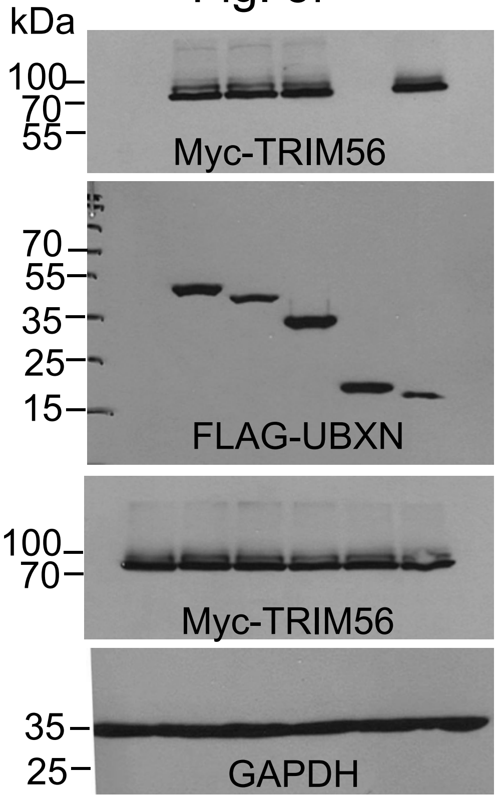

Fig. 6a

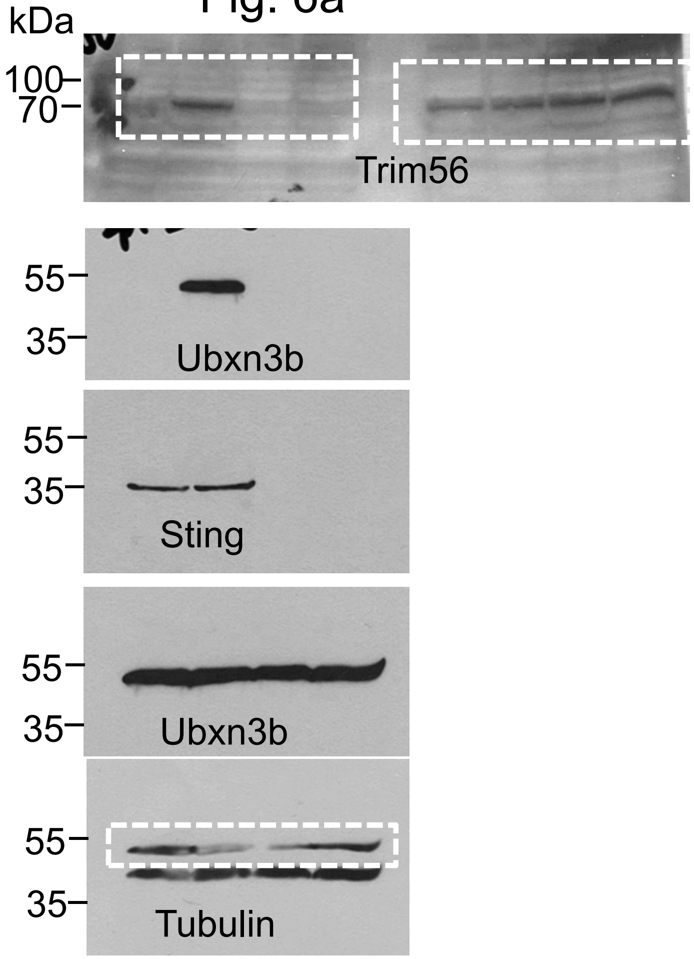

Fig. 6b

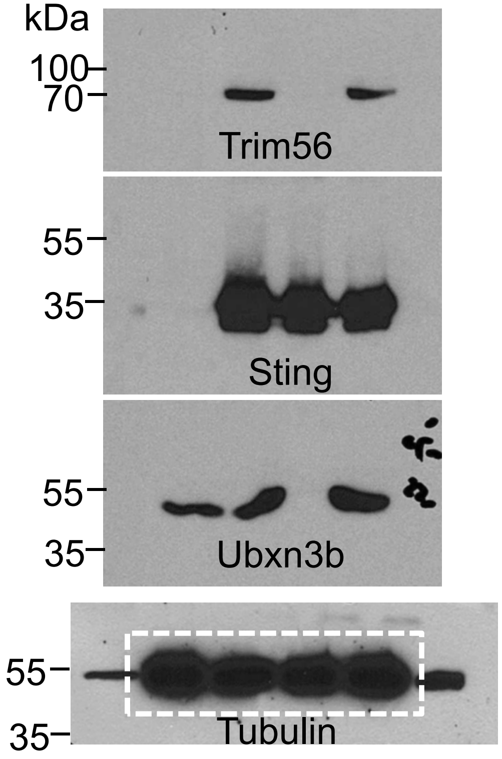

Fig. 6d

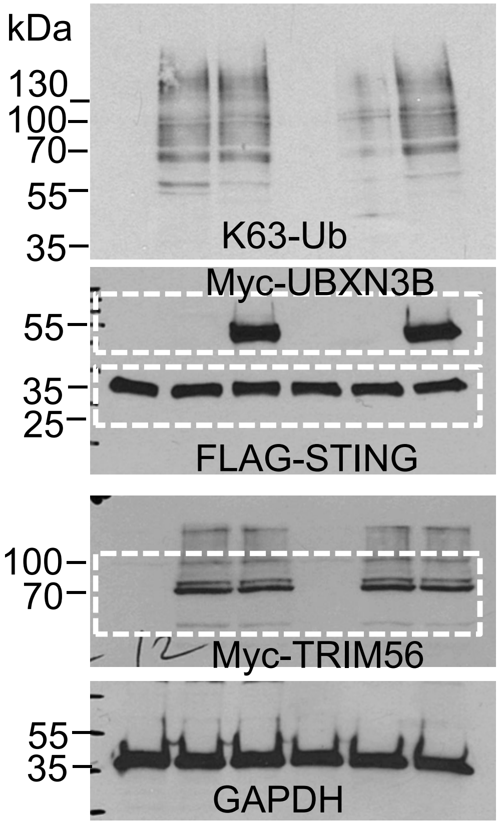

Fig. 6c

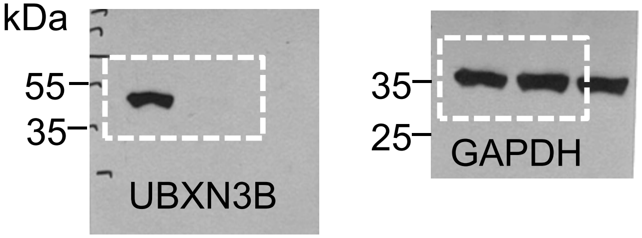

Fig. 6e

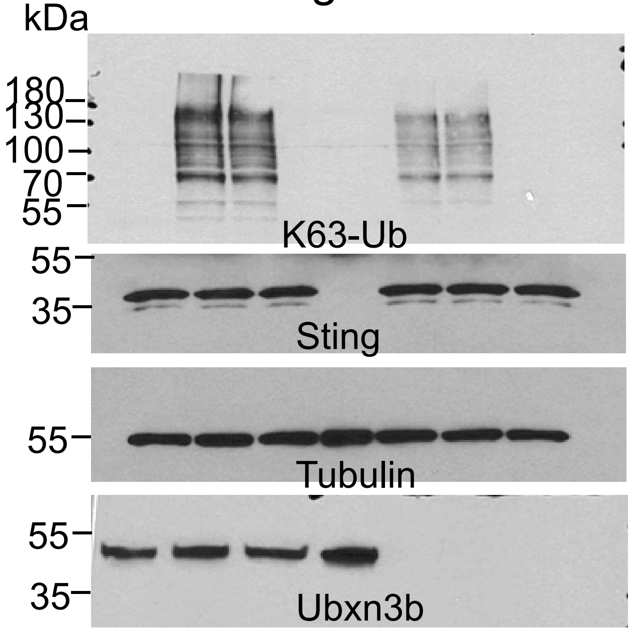

Supplementary Fig. 1a

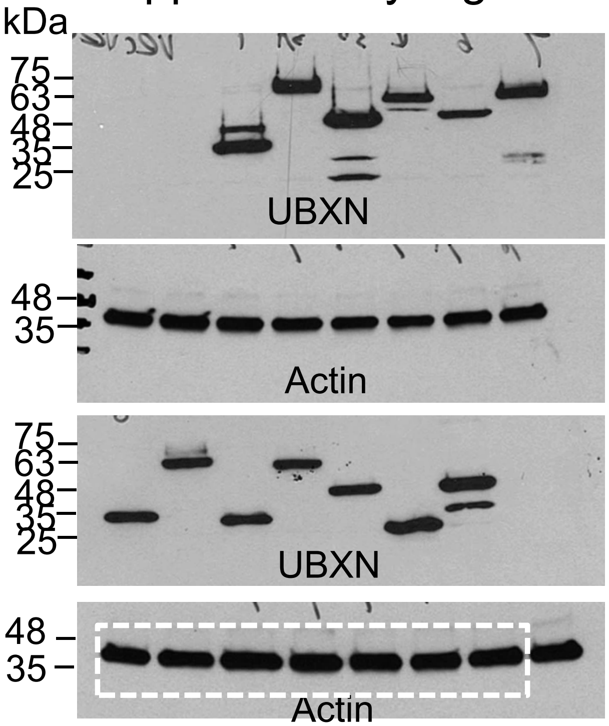

Supplementary Fig. 7

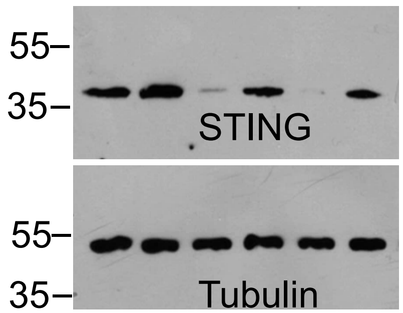

Supplementary Fig. 4a

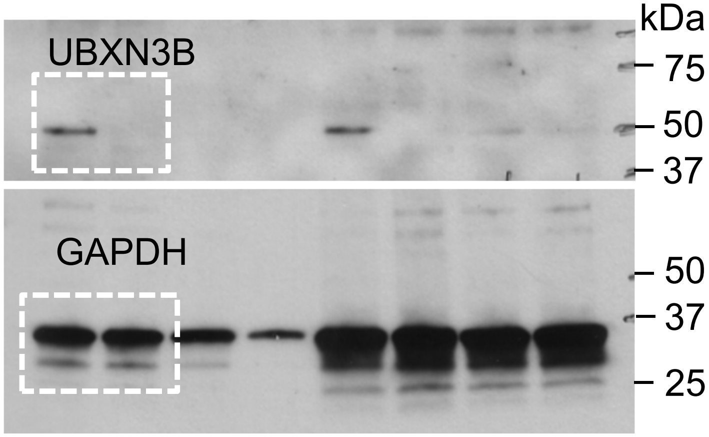

Supplementary Fig. 4d

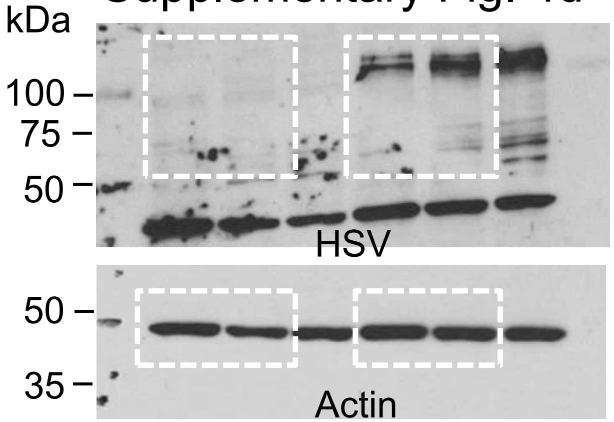

Supplementary Fig. 9b

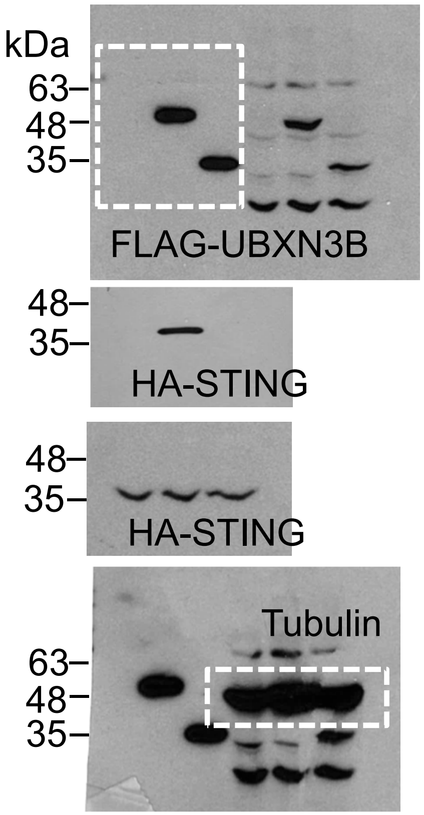

Supplementary Fig. 9c

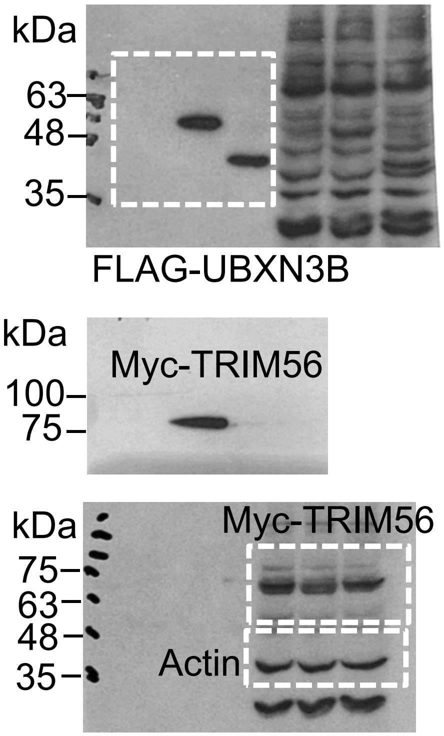

Supplementary Fig. 11a

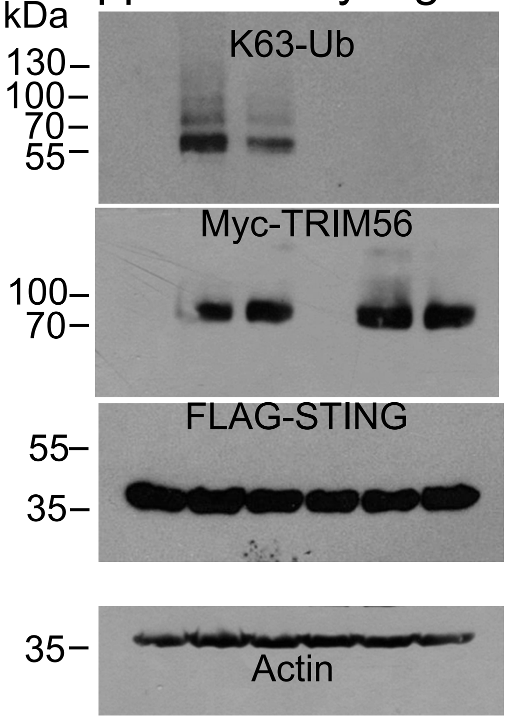

Supplementary Fig. 11b

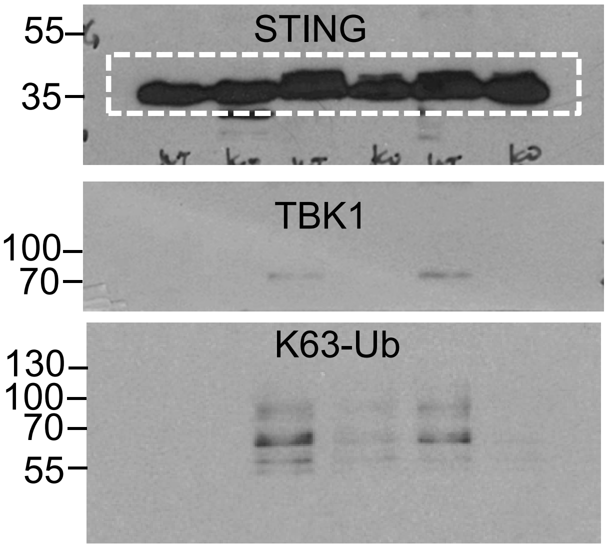

Supplementary Fig. 10

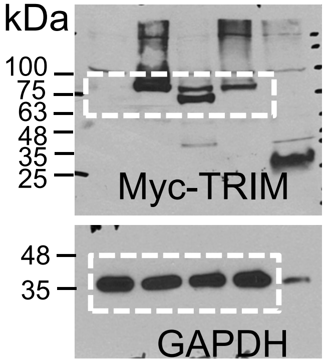

Supplement: Supplementary file 1 — Supplementary Information [file 41467_2018_4759_MOESM1_ESM.pdf]
