# Supplementary material for: UBXN3B positively regulates STING-mediated antiviral immune responses
Source: Nat Commun. 2018 Jun 13;9:2329. doi: 10.1038/s41467-018-04759-8 (PMC5998066; doi:10.1038/s41467-018-04759-8)
Supplement: Supplementary file 3 — Description of Additional Supplementary Files [file 41467_2018_4759_MOESM3_ESM.pdf]

Description of Additional Supplementary Files

File Name: Supplementary Movie 1
Description: This video depicts the neurological symptoms of <i>Ubxn3b</i>^{+/+} and <i>Ubxn3b</i>^{-/-} mice 3 days after HSV-1 infection.
